# Residential particulate matter and distance to roadways in relation to mammographic density: results from the Nurses’ Health Studies

**DOI:** 10.1186/s13058-017-0915-5

**Published:** 2017-11-23

**Authors:** Natalie C. DuPre, Jaime E. Hart, Kimberly A. Bertrand, Peter Kraft, Francine Laden, Rulla M. Tamimi

**Affiliations:** 1 Department of Epidemiology, Harvard T.H. Chan School of Public Health, Boston, MA USA; 2 Channing Division of Network Medicine, Department of Medicine, Brigham and Women’s Hospital and Harvard Medical School, Boston, MA USA; 3 Department of Environmental Health, Harvard T.H. Chan School of Public Health, Boston, MA USA; 4 Slone Epidemiology Center at Boston University, Boston, MA USA

**Keywords:** Air pollution, Particulate matter, Mammographic density, Epidemiology

## Abstract

**Background:**

High mammographic density is a strong, well-established breast cancer risk factor. Three studies conducted in various smaller geographic settings reported inconsistent findings between air pollution and mammographic density. We assessed whether particulate matter (PM) exposures (PM_2.5_, PM_2.5–10_, and PM_10_) and distance to roadways were associated with mammographic density among women residing across the United States.

**Methods:**

The Nurses’ Health Studies are prospective cohorts for whom a subset has screening mammograms from the 1990s (interquartile range 1990–1999). PM was estimated using spatio-temporal models linked to residential addresses. Among 3258 women (average age at mammogram 52.7 years), we performed multivariable linear regression to assess associations between square-root-transformed mammographic density and PM within 1 and 3 years before the mammogram. For linear regression estimates of PM in relation to untransformed mammographic density outcomes, bootstrapped robust standard errors are used to calculate 95% confidence intervals (CIs). Analyses were stratified by menopausal status and region of residence.

**Results:**

Recent PM and distance to roadways were not associated with mammographic density in premenopausal women (PM_2.5_ within 3 years before mammogram β = 0.05, 95% CI –0.16, 0.27; PM_2.5–10_ β = 0, 95%, CI –0.15, 0.16; PM_10_ β = 0.02, 95% CI –0.10, 0.13) and postmenopausal women (PM_2.5_ within 3 years before mammogram β = –0.05, 95% CI –0.27, 0.17; PM_2.5–10_ β = –0.01, 95% CI –0.16, 0.14; PM_10_ β = –0.02, 95% CI –0.13, 0.09). Largely null associations were observed within regions. Suggestive associations were observed among postmenopausal women in the Northeast (*n* = 745), where a 10-μg/m^3^ increase in PM_2.5_ within 3 years before the mammogram was associated with 3.4 percentage points higher percent mammographic density (95% CI –0.5, 7.3).

**Conclusions:**

These findings do not support that recent PM or roadway exposures influence mammographic density. Although PM was largely not associated with mammographic density, we cannot rule out the role of PM during earlier exposure time windows and possible associations among northeastern postmenopausal women.

**Electronic supplementary material:**

The online version of this article (doi:10.1186/s13058-017-0915-5) contains supplementary material, which is available to authorized users.

## Background

In 2013, 66.8% of women in the United States aged ≥ 40 years had undergone a mammogram within the previous 2 years [1]. Mammograms not only aid in breast tumor detection but also provide a radiographic image of the breast that distinguishes fat and fibroglandular tissue based on their differences in X-ray absorption. The fat tissue in the breast is radiolucent and appears dark on the mammogram, while the dense stromal and epithelial tissue is radiopaque and appears bright. The proportion of dense tissue area compared to the total breast tissue area yields a measure of percent breast density, which is an established breast cancer risk factor. In a meta-analysis, compared to women with less than 5% dense tissue on a mammogram, women with 50–74% dense tissue had a 3.0-fold increased risk of developing breast cancer and women with ≥ 75% dense tissue had a 4.6-fold increased risk [2]. Percent mammographic density was reported to be an intermediate marker of breast cancer risk for certain exposures, such as early life body size and hormonal therapy use, although not all breast cancer risk factors are mediated by mammographic density [3]. Therefore, identifying predictors of breast density variation may be important for breast cancer risk reduction and is becoming increasingly relevant in the United States as more than half of the states mandate that physicians notify women who have dense breasts. While many studies of breast density highlight the relevance of age, hormonal, and reproductive factors [4], limited research investigates the role of air pollutants that can bind to estrogen receptors to induce hormonal changes via endocrine disruption [5, 6].

The World Health Organization’s International Agency for Research on Cancer classified ambient air pollution and particulate matter (PM) as Group 1 human carcinogens largely based on results from lung cancer studies [7]. However, studies of air pollution metrics and risk of breast cancer have produced inconsistent results [8–16] and have been largely null for recent PM exposures in cohort studies [17–19], but this does not rule out whether PM in early time windows of exposure influences disease incidence or whether PM influences earlier changes in the breast. In 2012, the Institute of Medicine issued a report calling on breast cancer research to address early mechanisms of breast carcinogenesis as well as the influence of environmental exposures and pollutants across the life course [20]; studying the associations of pollutants and mammographic density variation can provide insight into early breast tissue variation that may translate to breast cancer risk reduction strategies. To date, three studies conducted in smaller geographic settings within Europe and the United States assessed associations between air pollution metrics and mammographic density measures but reported inconsistent findings [21–23], likely due to differences and limitations in PM measurement methods and the use of categorical measures of mammographic density.

Given the large geographical scope of the nationwide US-based Nurses’ Health Study (NHS) and NHSII cohorts and the ability to control for well-established breast cancer risk factors and predictors of mammographic density, we investigated associations between PM exposures at one’s residential address and distance to roadways in relation to a continuous measure of mammographic density in women without breast cancer. We assessed the associations separately for premenopausal and postmenopausal women and within regions of the United States.

## Methods

### Study population

The NHS cohort was established in 1976, enrolling 121,700 married, female US nurses between the ages of 30 and 55 years who resided in 11 states at entry (California, Connecticut, Florida, Massachusetts, Maryland, Michigan, New Jersey, New York, Ohio, Pennsylvania, and Texas) [24]. The NHSII cohort was established in 1989, enrolling 116,430 female US nurses between the ages of 25 and 42 years residing in 14 states (California, Connecticut, Indiana, Iowa, Kentucky, Massachusetts, Michigan, Missouri, New York, North Carolina, Ohio, Pennsylvania, South Carolina, and Texas). Briefly, participants in both cohorts completed baseline and subsequent biennial questionnaires on medical history and covariate data (e.g., anthropometrics, reproductive history, and lifestyle factors) that were mailed to their residential addresses. Participants reported breast cancer diagnoses on biennial questionnaires and the diagnoses were confirmed by review of medical records. In 1989–1990 and 1996–1999 a subset of participants from the NHS and the NHSII, respectively, provided blood samples, and breast cancer case–control studies nested within the NHS (cases *n* = 5371, controls *n* = 7469) and the NHSII (cases *n* = 2750, controls *n* = 5500) were established to evaluate blood-based biomarkers of risk. Around the time of blood draw, the majority of the NHS (80.1%) and NHSII (89.2%) participants reported having a mammogram in the last 2 years on the 1990 and 1999 questionnaires, respectively. Film mammograms were collected from women who were participants in the nested breast cancer case–control studies and who also reported mammography around the time of blood collection. We successfully obtained mammograms from approximately 80% of the eligible participants from the NHS (cases *n* = 1304, controls *n* = 2362) and the NHSII (cases *n* = 758, controls *n* = 1833) [25, 26]. These mammograms were primarily conducted in the early 1990s for the NHS (interquartile range (IQR) 1990–1994) and in the late 1990s for the NHSII (IQR 1997–2000). For this study, we used only mammograms from the controls that were reported as screening mammograms; mammograms for diagnostic purposes and those from breast cancer cases were excluded. Among the controls, women with and without collected mammograms were similar with regards to breast cancer risk factors such as age, body mass index, parity, and family history of breast cancer [25, 26].

### Outcomes: mammographic density measures

Film mammograms of the cranio-caudal views of each breast were digitized with a Lumysis 85 laser film scanner for all NHS mammograms and for the first two batches of NHSII mammograms, and with a VIDAR CAD PRO Advantage scanner (VIDAR Systems Corporation, Herndon, VA, USA) for the third batch of NHSII mammograms. The correlation between percent density measures from the two scanners was 0.88 [27]. Trained observers were blinded to exposure status and used a computer-assisted thresholding method (Cumulus software) to measure mammographic density. Breast density measurements were averaged across both breasts. Replicate mammograms from each batch of density readings exhibited high within-person intraclass correlation coefficients ≥ 0.90 [28]. Despite these high within-person intraclass correlation coefficients, between-batch variability was present in the NHSII. Batch adjustment methods were applied to the second and third NHSII batch measurements to reflect the density measurements had they been evaluated in the first batch [26, 29]. The primary outcome of interest was percent mammographic density (i.e., the dense area divided by the total breast area), and secondary outcomes of interest included absolute dense area (cm^2^) and absolute nondense area (cm^2^).

### Exposures: particulate matter and proximity to roadways

Residential addresses were updated biennially in both the NHS and the NHSII as part of the questionnaire mailing process. By the mid-1990s, participants resided in all 50 states [30]. Study participants’ residential addresses were geocoded and linked to predicted estimates of PM and to proximity to various-sized roadways over the course of the study.

i. Particulate matter
  In the current study, the primary exposures of interest were PM levels 1 year before the year of the mammogram and the cumulative average PM for up to 3 years prior to the mammogram year. Particulate matter is classified into three size fractions, including fine particles less than 2.5 μm in aerodynamic diameter (PM_2.5_), thoracic particles less than 10 μm (PM_10_), and coarse particles between 2.5 and 10 μm (PM_2.5–10_). Particulate matter comes from various sources and the varying contribution of these sources is different in each region of the United States. These sources generally include motor vehicle emissions, tire fragments, road dust, industrial and agricultural combustion, wood burning, pollens and molds, forest fires, volcanic emissions, and sea spray [31]. We defined the regions of the United States based on the Census Bureau designated regions [32]: Northeast (CT, ME, MA, NH, NJ, NY, PA, RI, and VT), Midwest (IL, IN, IA, KS, MI, MN, MO, NE, ND, OH, SD, and WI), South (AL, AR, DE, DC, FL, GA, KY, LA, MD, MS, NC, OK, SC, TN, TX, VA, and WV), and West (AZ, CA, CO, ID, MT, NV, NM, OR, UT, WA, and WY).
  Briefly, predictions of ambient PM were available across the 48 conterminous United States (i.e., all states excluding Alaska and Hawaii). Predictions of monthly average PM_2.5_ and PM_10_ were generated using geographic information system (GIS)-based spatio-temporal models to account for spatial and meteorological variation over time [33]. The monthly estimates were linked with participants’ residential addresses between 1988 and 2007 [33]. PM_2.5_ data were not directly measured before 1999, and therefore we derived PM_2.5_ levels before 1999 from the PM_10_ levels before 1999 using the 1999 ratio of PM_2.5_:PM_10_ [33]. Coarse particulate matter (PM_2.5–10_) was calculated as the difference between PM_10_ and PM_2.5_ estimates. The models were evaluated for predictive accuracy using a 10-set cross-validation approach; cross-validation correlation coefficients were high for PM_2.5_ (*R* ^2^ = 0.77) and moderate for PM_10_ (*R* ^2^ = 0.58) and PM_2.5–10_ (*R* ^2^ = 0.46) [33].
ii. Proximity to roadways
  Secondary exposures of interest included proximity between one’s residential address in the year before the mammogram to various types of major roadways as a proxy for traffic-related exposures. For women with a street-segment level geocoded address, proximity of residential address to nearest roadways was calculated in meters using GIS software and the ESRI StreetMap Pro 2007 road network data. Distances to three different types of roadways were classified based on the US Census Feature Class Code road classification system. The types of roadways included: A1 (primary roads, typically interstate highways, with limited access, division between opposing directions of traffic, and defined exits), A2 (primary major, noninterstate highways and major roads without access restrictions), and A3 (smaller, secondary roads, usually with more than two lanes).

### Study sample

To study the influence of PM and proximity to road on normal breast tissue composition, we restricted the analyses to NHS and NHSII participants without breast cancer for whom mammographic density data were available (i.e., controls within the original nested case–control studies; *n* = 2362 for NHS, *n* = 1833 for NHSII). Our analyses were further restricted to women who had a screening film mammogram dated between January 1990 and December 2008 and those with data available on estimated PM exposures living in the conterminous United States (*n* = 1821 for NHS, *n* = 1815 for NHSII). Because menopause status and BMI are the strongest predictors of mammographic density, we excluded women with missing or dubious menopause status (*n* = 308) or with missing BMI data (*n* = 70) at the time of mammogram. The final study sample comprised 3258 participants (*n* = 1624 premenopausal women; *n* = 1634 postmenopausal women).

### Covariates

We considered the following variables in the multivariable-adjusted models: cohort/batch (NHS first batch, NHS second batch, NHSII), age at mammogram (years), BMI at mammogram (kg/m^2^), categories of parity and age at first birth (nulliparous, 1–4 children and age at first birth < 25 years, 1–4 children and age at first birth 25–29 years, 1–4 children and age at first birth ≥ 30 years, ≥ 5 children, missing), categories of hormonal therapy use (never, current, past, missing), history of biopsy-confirmed benign breast disease, age at menopause (for postmenopausal women only), categories of breast feeding status (nulliparous, never breast fed, ever breast fed, missing), age at menarche (years), oral contraceptive use among premenopausal women only (not on oral contraceptives, current for < 5 years, current for 5 + years, missing), family history of breast cancer, race/ethnicity (White, Black, Hispanic, Other), BMI at age 18 (<19 kg/m^2^, 19–24.9 kg/m^2^, 25–29.9 kg/m^2^, ≥ 30 kg/m^2^, missing), alcohol consumption (g/day), physical activity (MET hours/week), Census tract-level median house value and median income based on values from the 2000 Census, region of residence (Northeast, Midwest, West, South), and date of mammogram to account for the strong decreasing trends in PM levels over time.

### Statistical analyses

Analyses were carried out separately for women who were premenopausal and postmenopausal at the time of the mammogram. Mammographic density measures were square-root transformed to achieve the statistical assumptions for linear regression. Multivariable linear regression was conducted to estimate the average difference in square-root-transformed breast density measures for a 10-μg/m^3^ increase of PM_2.5_, PM_2.5–10_, and PM_10_ and to compare previously published categories of residential proximity to A1, A1–A2, and A1–A3 roadways (< 50 meters, 50–199 meters, and ≥ 200 meters as the referent group) [30]. In the final multivariable models for premenopausal and postmenopausal women, we included the strongest predictors of mammographic density including cohort/batch, age at mammogram, BMI at mammogram, parity and age at first birth categories, hormonal therapy use, and history of biopsy-confirmed benign breast disease. In sensitivity analyses, we additionally considered other aforementioned covariates. When we present the linear regression estimates for the untransformed mammographic density outcome measures as the dependent variable, bootstrapped robust standard errors are used to calculate 95% confidence intervals (CIs).

We used the likelihood ratio test (LRT) to determine whether the associations between the exposures and percent density varied by cohort and by region of residence. In addition to the PM results presented across the entire United States, estimates from multivariable models were presented separately for each region. We used nonparametric restricted cubic regression splines [34] to determine whether the associations between PM and percent mammographic density were nonlinear. In sensitivity analyses, we additionally restricted the sample to women who did not move to another state before their mammogram.

All analyses were conducted in SAS version 9.4 (SAS, Cary, NC, USA).

## Results

The 1989 median level of PM_2.5_ was 16.9 μg/m^3^ (IQR 14.4, 19.5), of PM_2.5–10_ was 10.6 μg/m^3^ (IQR 8.1, 14.2), and of PM_10_ was 27.7 μg/m^3^ (IQR 23.8, 32.2). The highest median PM_2.5_ level was in the Midwest (18.5 μg/m^3^, IQR 16.2, 20.5) followed by the Northeast (16.6 μg/m^3^, IQR 14.7, 18.9), the West (15.5 μg/m^3^, IQR 13.4, 20.8), and the South (13.0 μg/m^3^, IQR 11.0, 17.8). The highest median PM_2.5–10_ level was in the West (19.6 μg/m^3^, IQR 16.4, 25.3) followed by the South (12.4 μg/m^3^, IQR 10.8, 13.9), the Midwest (10.8 μg/m^3^, IQR 8.3, 14.3), and the Northeast (8.6 μg/m^3^, IQR 7.1, 10.3). There were no statistically significant interactions with PM exposures and cohort for the multivariable models among premenopausal or among postmenopausal women (LRT *p* > 0.15).

### Premenopausal women

Premenopausal women (*n* = 1624) in the highest year-adjusted quintiles of PM_2.5_ 1 year before the mammogram were more likely to be overweight/obese at age 18, to have never breast fed, to be current oral contraceptive users, and were less likely to have a history of benign breast disease or family history of breast cancer compared to women in the lowest quintile (Table 1).

**Table 1:** Age-standardized characteristics by calendar-year-adjusted PM_2.5_ quintiles in premenopausal (*n* = 1624) and postmenopausal (*n* = 1634) participants

	Premenopausal women		Postmenopausal women	
Characteristic^a^	1st quintile (*n* = 331)	5th quintile (*n* = 332)	1st quintile (*n* = 331)	5th quintile (*n* = 334)
Age at mammogram^b^	45.6 ± 4.2	45.6 ± 4.3	60.0 ± 7.4	59.7 ± 7.6
NHSII cohort (%)	76	78	19	24
BMI at mammogram (kg/m^2^)	25.1 ± 5.6	25.8 ± 5.5	26.1 ± 5.0	26.5 ± 5.6
Presence of biopsy-confirmed benign breast disease (%)	18.8	17.8	23.4	21.8
Current use of hormonal therapy (%)	2.7	2.3	52.2	47.3
Parity and age at first birth status (%)				
Nulliparous	16.3	16.5	9.8	9.8
1–4 children before age 25	29.0	29.3	36.5	38.2
1–4 children between age 25 and 30	33.8	34.4	31.9	29.5
1–4 children after age 30	17.7	17.0	8.6	9.8
5+ children	2.6	1.9	12.6	11.7
Missing	0.6	0.9	0.5	1.0
Categories of BMI at age 18 (%)				
< 19.0 kg/m^2^	16.0	17.8	10.6	13.3
19.0–24.9 kg/m^2^	72.2	70.7	75.6	72.9
25–29.9 kg/m^2^	7.4	8.5	9.4	8.1
30+ kg/m^2^	1.9	1.7	0.9	1.9
Missing	2.5	1.3	3.5	3.8
Family history of breast cancer (%)	10.7	8.5	13.0	11.0
Age at menarche (years)	12.4 ± 1.4	12.4 ± 1.4	12.4 ± 1.3	12.6 ± 1.5
Lactation among parous women (%)				
Never breastfed	15.0	21.9	35.8	41.5
Yes‚ breastfed > 1 month	82.5	75.1	62.8	57.0
Missing	2.5	3.1	1.5	1.3
Oral contraceptive use (%)				
Not on oral contraceptives	94.4	93.4	–	–
Current < 5 years	2.6	1.5	–	–
Current 5 + years	2.7	4.9	–	–
Missing	0.3	0.3	–	–
Alcohol consumption (g/day)	5.3 ± 8.4	3.8 ± 6.3	5.6 ± 9.1	4.8 ± 8.1
Physical activity (MET hours/week)	22.2 ± 29.6	17.5 ± 19.2	17.6 ± 17.3	19.4 ± 26.3
White (%)	99.4	97.5	98.6	97.7
Region of residence (%)				
Northeast	27.8	27.4	28.0	33.4
Midwest	13.8	40.5	6.5	34.8
West	39.4	16.5	33.8	20.6
South	18.9	15.6	31.7	11.2
Distance from residential address to A1–A3 roadways (meters)	313.8 ± 185.9	231.1 ± 169.6	299.0 ± 187.5	220.2 ± 168.1

*PM*_*2.5*_ particulate matter less than 2.5 μm in diameter, *NHSII* Nurses’ Health Study II, *BMI* body mass index, *MET* metabolic equivalent

^a^Values are mean ± SD or percentages and are standardized to the age distribution of the study population. Values of polytomous variables may not sum to 100% due to rounding

^b^Value is not age adjusted

Among premenopausal women residing across the United States, no associations were observed between PM exposures and percent mammographic density (Table 2), dense area, or nondense area (see Additional file 1) after multivariable adjustment. The patterns of association between PM and percent density were similar after further adjustment for other covariates and after restricting to women who did not move to another state before the mammogram. The associations were null and not statistically significant comparing premenopausal women who live closer to roadways to those who live further away (Table 3). There was no evidence for a nonlinear relationship between PM and percent mammographic density.

**Table 2:** Estimated differences^a^ (95% confidence interval) in square-root-transformed percent mammographic density for a 10-μg/m^3^ PM increase

	Across the United States	Northeast	Midwest	West	South	*p* for region interaction term
Premenopausal						
1 year prior	*n* = 1624	*n* = 577	*n* = 554	*n* = 261	*n* = 232	
PM_2.5_	0.04 (–0.17, 0.26)	0.06 (–0.40, 0.52)	–0.08 (–0.58, 0.43)	0 (–0.35, 0.34)	0.21 (–0.32, 0.74)	0.59
PM_2.5–10_	–0.01 (–0.16, 0.15)	–0.01 (–0.57, 0.54)	–0.07 (–0.42, 0.28)	0.08 (–0.19, 0.34)	–0.34 (–0.95, 0.27)	0.45
PM_10_	0.01 (–0.10, 0.12)	0.02 (–0.26, 0.30)	–0.06 (–0.33, 0.20)	0.03 (–0.14, 0.20)	–0.05 (–0.57, 0.47)	0.58
3-year average	*n* = 1624	*n* = 582	*n* = 554	*n* = 260	*n* = 228	
PM_2.5_	0.05 (–0.16, 0.27)	0.05 (–0.40, 0.51)	–0.05 (–0.55, 0.45)	–0.01 (–0.36, 0.35)	0.27 (–0.27, 0.81)	0.53
PM_2.5–10_	0 (–0.15, 0.16)	0.02 (–0.53, 0.57)	–0.04 (–0.40, 0.33)	0.10 (–0.18, 0.38)	–0.58 (–1.19, 0.03)	0.22
PM_10_	0.02 (–0.10, 0.13)	0.02 (–0.25, 0.30)	–0.03 (–0.30, 0.23)	0.04 (–0.13, 0.20)	–0.17 (–0.70, 0.36)	0.67
Postmenopausal						
1 year prior	*n* = 1634	*n* = 738	*n* = 360	*n* = 274	*n* = 262	
PM_2.5_	–0.04 (–0.26, 0.19)	0.37 (–0.03, 0.77)	–0.24 (–0.91, 0.42)	–0.21 (–0.56, 0.13)	–0.05 (–0.70, 0.60)	0.09
PM_2.5–10_	–0.01 (–0.16, 0.14)	0.22 (–0.24, 0.69)	0.11 (–0.38, 0.60)	–0.22 (–0.47, 0.03)	0.32 (–0.32, 0.96)	0.29
PM_10_	–0.01 (–0.12, 0.10)	0.19 (–0.05, 0.42)	–0.01 (–0.34, 0.32)	–0.14 (–0.29, 0.02)	0.19 (–0.35, 0.74)	0.15
3-year average	*n* = 1634	*n* = 745	*n* = 364	*n* = 272	*n* = 253	
PM_2.5_	–0.05 (–0.27, 0.17)	0.39 (–0.02, 0.79)	–0.25 (–0.91, 0.42)	–0.27 (–0.62, 0.07)	0.06 (–0.61, 0.73)	0.06
PM_2.5–10_	–0.01 (–0.16, 0.14)	0.19 (–0.27, 0.65)	0.16 (–0.32, 0.64)	–0.23 (–0.47, 0.02)	0.39 (–0.30, 1.07)	0.24
PM_10_	–0.02 (–0.13, 0.09)	0.18 (–0.06, 0.41)	0.02 (–0.31, 0.34)	–0.15 (–0.31, 0.01)	0.31 (–0.26, 0.89)	0.10

*PM* particulate matter, *PM*_*2.5*_ particulate matter less than 2.5 μm in diameter, *PM*_*2.5–10*_ particulate matter between 2.5 and 10 μm in diameter, *PM*_*10*_ particulate matter less than 10 μm in diameter

^a^Adjusted for cohort, age, body mass index, parity and age at first birth categories, hormonal therapy use, history of biopsy-confirmed benign breast disease, and date of mammogram

**Table 3:** Estimated differences^a^ (95% confidence interval) in square-root-transformed percent mammographic density by distance to roadways.

	*n*	Premenopausal	*p* for region interaction term	*n*	Postmenopausal	*p* for region interaction term
Distance to A1 roads						
≥ 200 meters	1576	Referent	0.95	1598	Referent	0.001
< 200 meters	47	–0.25 (–0.65, 0.15)		36	0.10 (–0.39, 0.60)	
*p* value		0.22			0.68	
Distance to A1–A2 roads			0.90			0.48
≥ 200 meters	1500	Referent		1510	Referent	
50–199 meters	87	–0.13 (–0.43, 0.16)		84	–0.03 (–0.35, 0.30)	
< 50 meters	36	0.06 (–0.39, 0.51)		40	–0.12 (–0.59, 0.35)	
*p* trend		0.65			0.88	
Distance to A1–A3 roads			0.43			0.47
≥ 200 meters	978	Referent		936	Referent	
50–199 meters	413	–0.05 (–0.21, 0.11)		431	–0.16 (–0.33, 0.01)	
< 50 meters	232	0.03 (–0.17, 0.23)		267	–0.11 (–0.31, 0.10)	
*p* trend		0.73			0.17	

^a^Adjusted for cohort, age at mammogram, body mass index at mammogram, parity and age at first birth categories, hormonal therapy use, history of biopsy-confirmed benign breast disease, and date of mammogram

### Postmenopausal women

Postmenopausal women (*n* = 1634) in the highest year-adjusted quintiles of PM_2.5_ 1 year before the mammogram were similar in terms of most mammographic density predictors compared to women in the lowest quintile (Table 1). However, women in the highest quintile were more likely to have never used hormonal therapy or lactated and were less likely to have had a history of benign breast disease and family history of breast cancer compared to women in the lowest quintile.

There were no associations between PM exposures or proximity to roadways and mammographic density outcomes for postmenopausal women overall (Tables 2 and 3; see Additional file 1: Table S1 for dense and nondense area outcomes). We did observe borderline statistically significant interactions between region and PM_2.5_ and PM_10_ levels with percent density (Table 2, *p* for interaction with region < 0.10). Among postmenopausal women in the Midwest and the South, the results were null and not statistically significant for PM exposures and density measures (Table 2; see Additional file 1: Table S1 for dense and nondense area outcomes).

Among postmenopausal women in the West, there were no statistically significant associations between PM_2.5_ and transformed mammographic density measures; however, suggestive inverse associations were observed for PM_2.5–10_ with percent density (β = –0.23, 95% CI –0.47, 0.02; Table 2), although the *p* value for interaction between PM_2.5–10_ and region was not statistically significant (*p* for interaction = 0.24). For the estimates of untransformed percent mammographic density, a 10-μg/m^3^ increase in cumulative PM_2.5–10_ up to 3 years before the mammogram was associated with an average difference of –2.0 percentage points (95% CI –4.7, 0.6; Additional file 1: Table S2). The patterns of association for postmenopausal PM results in the West did not change meaningfully after additional adjustment of other covariates or after restricting to nonmovers.

In contrast among postmenopausal women in the Northeast, there were no statistically significant associations between PM_2.5–10_ and transformed mammographic density measures (Table 2; see Additional file 1: Table S1 for dense and nondense area measures); however, there were suggestive positive associations between PM_2.5_ and transformed percent mammographic density (β = 0.39, 95% CI –0.02, 0.79; Table 2) and significant inverse associations with transformed nondense area (β = –0.76, 95% CI –1.41, –0.11; see Additional file 1: Table S1). For the estimates of untransformed mammographic density measures, a 10-μg/m^3^ increase in 3-year cumulative PM_2.5_ in the Northeast was associated with an average difference in percent mammographic density of 3.4 percentage points (95% CI –0.5, 7.3; Additional file 1: Table S2) and an average difference in nondense area of –17.2 cm^2^ (95% CI –36.1, –0.5; Additional file 1: Table S2). The associations did not change meaningfully after further adjustment for other covariates or after restricting to nonmovers. There was no evidence for a nonlinear relationship between PM and percent mammographic density.

## Discussion

In summary, we observed no overall association between residential PM exposures or proximity to roadways and mammographic density in premenopausal and postmenopausal women residing across the conterminous United States. This is consistent with the reported null overall associations between PM and breast cancer incidence in prospective cohort studies from the Danish Nurse Cohort, the NHSII, and the Sister Study Cohort [17–19]. Upon further exploration, we did observe differences in associations with PM exposures and mammographic density by region of the United States among postmenopausal women. Recent exposure to fine particulate matter (PM_2.5_) in the Northeast was associated with a percent mammographic density 3.4 percentage points higher percent mammographic density (95% CI –0.5, 7.3) among postmenopausal women. Unexpectedly, recent coarse particulate matter (PM_2.5–10_) in the West showed a weaker, inverse association with percent density (–2.0 percentage point difference, 95% CI –4.7, 0.6).

Three studies based in Denmark, in the Netherlands, and in five registries in the US Breast Cancer Surveillance Consortium (New Hampshire, Vermont, New Mexico, San Francisco, and western Washington) reported inconsistent findings between air pollution exposures and mammographic density [21–23], potentially due to different measurements and distributions of mammographic density and air pollution. In Denmark [22], the authors found a weak inverse association between having mixed/dense breasts and residential exposure to nitrogen oxide that did not differ by menopausal status, but did not assess PM or continuous measures of mammographic density. In the Dutch study of primarily postmenopausal women [21], there were small positive associations observed between continuous percent mammographic density and residential nitrogen dioxide and PM_2.5_ absorbance but null associations for small increases in PM_2.5_, PM_2.5–10_, and PM_10_ exposures. In the five registries in the US-based Breast Cancer Surveillance Consortium [23], PM_2.5_ at the participants’ zip code was positively associated with categorical measures of mammographic density based on the American College of Radiology’s Breast Imaging-Reporting and Data System (BI-RADS) categories. In addition to the methodologic differences in measurement of mammographic density and air pollution across previous studies, the inconsistent findings suggest that geospatial variation in air pollution composition may account for the different findings in Denmark, the Netherlands, and the United States. In the current study, we observed regional differences among postmenopausal women with positive associations between PM_2.5_ and mammographic density in the Northeastern United States, but null findings between PM_2.5_ and mammographic density in the Midwest, South, and West. Regional differences have also been noted in cardiovascular disease outcomes, with stronger associations with PM_2.5_ in the Northeast [35]. This may be in part due to the differences in PM_2.5_ levels, composition, and sources across regions in the United States.

Fine particles (PM_2.5_) are primarily from combustion sources, organic compounds, and metals that can penetrate the small airways and alveoli deep in the lung [31] and have an atmospheric half-life ranging from days to weeks [36]. In the United States, approximately 80% of PM_2.5_ composition consists of sulfates, nitrates, ammonium, elemental carbon, organic carbon, Na^+^, and silicon and the remaining ~ 20% is a catch-all category consisting largely of many minerals and metals (e.g., Pb, Cd, V, Ni, Cu, Zn, Mn, and Fe); however, the distribution of these major components differs across the United States [36, 37]. In the Eastern United States, the proportions of sulfate, ammonium, and the catch-all category for other constituents were higher than in the Western United States, with larger differences in summer, whereas PM_2.5_ composition in the Western United States was higher in organic carbon, elemental carbon, nitrates, and silicon [36, 37]. Furthermore, PM_2.5_ levels were highest in the Eastern United States [36, 37], particularly in the Northeast [37]. The EPA describes in detail the formation, composition, and sources of PM_2.5_ and PM_2.5–10_ [36]. The biological effects of exposure to PM appear to go beyond the lung inducing systemic inflammation, oxidative stress, and epigenetic changes seen with alterations in circulating C-reactive protein, fibrinogen, white blood cell counts, tumor necrosis factor alpha, interleukin-6, DNA adducts, protein, lipids and DNA oxidation [38], and DNA methylation [39, 40]. Taken together, the higher levels and more heterogeneous composition of PM_2.5_ in the Northeast than in other regions as well as the half-life and biological plausibility of PM_2.5_ to induce systemic changes may be germane to variation in breast tissue composition seen only with PM_2.5_ among postmenopausal women residing in the Northeast. Future studies of PM and postmenopausal breast cancer risk should be aware of the differences in PM composition across regions of the United States.

While we observed largely null PM_2.5–10_ associations, the inverse associations between coarse PM_2.5–10_ and percent mammographic density among postmenopausal women in the West were surprising, were lacking in biological plausibility, and were likely due to the greater uncertainty inherent in the PM_2.5–10_ measurements or chance. PM_2.5–10_ is formed by the break-up of large solids and droplets (e.g., crushing, grinding and abrasion of surfaces, dust suspension, and the evaporation of ocean sprays) and is largely composed of soil, street dust, fly ash from uncontrolled combustion, nitrates, sulfates, crustal oxides (Si, Al, and Fe), sea salt, pollen, fungal spores, insect fragments, other bioaerosols, and automobile debris. The atmospheric half-life of PM_2.5–10_ is shorter than that of PM_2.5_ ranging from minutes to hours and PM_2.5–10_ can penetrate the extrathoracic and upper tracheobronchial regions [36]. Compared to the PM_2.5_ measurement estimations, coarse PM_2.5–10_ estimates had lower cross-validation *R* ^2^ coefficients across all US regions (PM_2.5_ *R* ^2^ = 0.77 versus PM_2.5–10_ *R* ^2^ = 0.46) and within regions (PM_2.5_ in Southwest *R* ^2^ = 0.77, Northwest *R* ^2^ = 0.56, Northeast *R* ^2^ = 0.72 versus PM_2.5–10_ in Southwest *R* ^2^ = 0.53, Northwest *R* ^2^ = 0.54, Northeast *R* ^2^ = 0.32), which suggests that PM_2.5–10_ estimates had more error on average than PM_2.5_ estimates [33].

There are several limitations and strengths of the study. Exposure measurement error is often a challenge. Several types of error can contribute to measurement error of PM. Instead of collecting personal exposure data that are not feasible on a large epidemiologic scale, we used predictions from spatio-temporal modeling. These data are subject to both Berkson error, which results in imprecision, and classical error that usually results in attenuated estimates toward the null [41, 42]. A combination of these errors could be the reason for the largely null findings. Furthermore, PM has many constituents [43, 44] and while the complex mixture of constituents was largely not associated with mammographic density, one cannot rule out that certain constituents may have an effect that was not captured by our measures of PM. While we did observe suggestive subgroup findings among postmenopausal women, it is possible that chance may explain the subgroup findings. Another limitation of the study’s exposure assessment is the inability to incorporate time spent at the residence or the time spent exposed to outdoor air pollution at the residence, and data on other ambient air pollutants such as NO_x_/NO_2_ were not available in these cohorts. In this study, we used spatio-temporal PM modeling that can reduce classical error; this technique has been reported to be more strongly correlated with personal PM exposure than using PM values from a nearest monitor [41]. Lastly, we were able to investigate the relationship of recent PM exposures; however, recent environmental exposures may not be the most relevant time window of exposure as research is pointing to the importance of early life exposures around the time of puberty and a woman’s first birth [45–48]. In spite of the limitations inherent in the exposure assessment, the strengths of the study included using a model of PM estimates that have been associated with other health conditions in this cohort, including mortality, cardiovascular disease, lung cancer, hypertension, pulmonary embolism, and cognitive decline [30, 49–54]. Furthermore, the study was conducted among women residing across the conterminous United States, making it the most geographically expansive study of mammographic density and air pollution to date. The large size of the study allowed for stratified analyses to explore the associations separately for premenopausal and postmenopausal women and to assess regional variations in the associations of PM and mammographic density. Lastly, many of the known predictors of mammographic density were considered to control for potential confounding.

## Conclusions

This study does not provide evidence that PM in the United States is associated with breast density variation. However, there is suggestive evidence that fine PM in the Northeast United States may influence breast tissue composition for postmenopausal women. Furthermore, this study cannot rule out the potential relationship of PM exposures during earlier time windows of exposure and mammographic density.

## Additional file

Additional file 1: Table S1. Presenting adjusted estimates (95% CI) of the difference in square-root-transformed mammographic dense area and nondense area for a 10-μg/m^3^ increase in PM among premenopausal and postmenopausal women residing across the United States and within regions, and **Table S2.** presenting adjusted estimates (95% CI) of the difference in untransformed mammographic density measures for a 10-μg/m^3^ increase in PM using bootstrapped robust standard errors (DOCX 33 kb)

## Abbreviations

BI-RADS: Breast Imaging-Reporting and Data System
BMI: Body mass index
CI: Confidence interval
EPA: Environmental Protection Agency
GIS: Geographic information system
IQR: Interquartile range
LRT: Likelihood ratio test
NHS: Nurses’ Health Study
NHSII: Nurses’ Health Study II
PM: Particulate matter (PM_2.5_, PM_2.5–10_, and PM_10_)
