# Supplementary material for: Residential particulate matter and distance to roadways in relation to mammographic density: results from the Nurses’ Health Studies
Source: Breast Cancer Res. 2017 Nov 23;19:124. doi: 10.1186/s13058-017-0915-5 (PMC5701365; doi:10.1186/s13058-017-0915-5)
Supplement: Additional file 1: Table S1. — Presenting adjusted estimates (95% CI) of the difference in square-root-transformed mammographic dense area and nondense area for a 10-μg/m3 increase in PM among premenopausal and postmenopausal women residing across the United States and within regions, and Table S2. presenting adjusted estimates (95% CI) of the difference in untransformed mammographic density measures for a 10-μg/m3 increase in PM using bootstrapped robust standard errors (DOCX 33 kb) [file 13058_2017_915_MOESM1_ESM.docx]

**Additional file 1: Supplemental Material**

Residential Particulate Matter and Distance to Roadways in Relation to Mammographic Density: Results from the Nurses’ Health Studies

Natalie C. DuPre, Jaime E. Hart, Kimberly A. Bertrand, Peter Kraft, Francine Laden*, and Rulla M. Tamimi*

*indicates shared senior co-authors

**Table of Contents**

**Table S1.** Adjusted estimates (95% CI) of the difference in square-root transformed mammographic dense area and non-dense area for a 10 µg/m^3^ increase in PM among pre- and post-menopausal women residing across the U.S. and within regions

**Table S2.** Adjusted estimates (95% CI) of the difference in untransformed mammographic density measures for a 10 µg/m^3^ increase in PM using bootstrapped robust standard errors

**Table S1.** Adjusted^a^ estimates (95% CI) of the difference in square-root transformed mammographic dense area and non-dense area for a 10 µg/m^3^ increase in particulate matter among pre- and post-menopausal women residing across the U.S. and within regions

|  | Across the U.S. | Northeast | | Midwest | West | South | P for region interaction term |
| --- | --- | --- | --- | --- | --- | --- | --- |
| ***Dense-area*** | | | | | | | |
| ***Pre-menopausal*** |  |  | |  |  |  |  |
| 1-year prior | n=1,624 | n=577 | | n=554 | n=261 | n=232 |  |
| PM_2.5_ | 0.08 (-0.31, 0.46) | 0.10 (-0.72, 0.92) | | 0.24 (-0.67, 1.15) | 0.04 (-0.58, 0.66) | 0.06 (-0.87, 1.00) | 0.96 |
| PM_2.5-10_ | -0.18 (-0.45, 0.10) | 0.15 (-0.84, 1.14) | | -0.38 (-1.02, 0.25) | -0.14 (-0.61, 0.32) | -0.47 (-1.55, 0.60) | 0.83 |
| PM_10_ | -0.07 (-0.27, 0.13) | 0.07 (-0.42, 0.56) | | -0.15 (-0.63, 0.33) | -0.05 (-0.34, 0.25) | -0.28 (-1.20, 0.63) | 0.90 |
| 3-year average | n=1,624 | n=582 | | n=554 | n=260 | n=228 |  |
| PM_2.5_ | 0.10 (-0.29, 0.48) | 0.08 (-0.72, 0.89) | | 0.27 (-0.64, 1.18) | 0.07 (-0.57, 0.70) | 0.03 (-0.91, 0.98) | 0.96 |
| PM_2.5-10_ | -0.16 (-0.44, 0.12) | 0.15 (-0.83, 1.13) | | -0.28 (-0.94, 0.38) | -0.12 (-0.62, 0.38) | -0.71 (-1.78, 0.36) | 0.84 |
| PM_10_ | -0.06 (-0.26, 0.15) | 0.07 (-0.42, 0.55) | | -0.08 (-0.57, 0.41) | -0.03 (-0.33, 0.27) | -0.50 (-1.42, 0.42) | 0.96 |
| ***Post-menopausal*** |  |  | |  |  |  |  |
| 1-year prior | n=1,634 | n=738 | | n=360 | n=274 | n=262 |  |
| PM_2.5_ | -0.09 (-0.41, 0.24) | 0.21 (-0.39, 0.81) | | -0.56 (-1.55, 0.43) | -0.24 (-0.73, 0.25) | 0.19 (-0.75, 1.12) | 0.22 |
| PM_2.5-10_ | -0.14 (-0.36, 0.08) | 0.02 (-0.68, 0.72) | | 0.15 (-0.59, 0.88) | -0.29 (-0.64, 0.07) | 0.26 (-0.65, 1.18) | 0.59 |
| PM_10_ | -0.10 (-0.26, 0.07) | 0.08 (-0.28, 0.43) | | -0.07 (-0.57, 0.42) | -0.17 (-0.40, 0.06) | 0.32 (-0.46, 1.09) | 0.36 |
| 3-year average | n=1,634 | n=745 | | n=364 | n=272 | n=253 |  |
| PM_2.5_ | -0.08 (-0.41, 0.25) | 0.21 (-0.41, 0.83) | | -0.40 (-1.39, 0.59) | -0.27 (-0.76, 0.22) | 0.31 (-0.64, 1.26) | 0.22 |
| PM_2.5-10_ | -0.12 (-0.34, 0.10) | -0.05 (-0.75, 0.65) | | 0.33 (-0.38, 1.05) | -0.29 (-0.65, 0.06) | 0.56 (-0.41, 1.53) | 0.31 |
| PM_10_ | -0.09 (-0.25, 0.08) | 0.06 (-0.30, 0.41) | | 0.06 (-0.43, 0.54) | -0.18 (-0.40, 0.05) | 0.62 (-0.19, 1.43) | 0.19 |
| ***Non-dense area*** | | | | | | | |
| ***Pre-menopausal*** |  |  |  | |  |  |  |
| 1-year prior | n=1,624 | n=577 | n=554 | | n=261 | n=232 |  |
| PM_2.5_ | -0.16 (-0.53, 0.21) | -0.16 (-0.90, 0.57) | 0.41 (-0.45, 1.28) | | -0.14 (-0.78, 0.49) | -0.39 (-1.34, 0.56) | 0.23 |
| PM_2.5-10_ | -0.15 (-0.42, 0.11) | 0.15 (-0.74, 1.04) | -0.23 (-0.83, 0.38) | | -0.42 (-0.90, 0.06) | 0.56 (-0.54, 1.66) | 0.09 |
| PM_10_ | -0.13 (-0.32, 0.07) | -0.02 (-0.46, 0.42) | -0.01 (-0.47, 0.44) | | -0.20 (-0.50, 0.10) | 0.03 (-0.90, 0.97) | 0.25 |
| 3-year average | n=1,624 | n=582 | n=554 | | n=260 | n=228 |  |
| PM_2.5_ | -0.15 (-0.53, 0.22) | -0.13 (-0.85, 0.60) | 0.33 (-0.53, 1.20) | | -0.11 (-0.75, 0.53) | -0.58 (-1.55, 0.40) | 0.24 |
| PM_2.5-10_ | -0.17 (-0.44, 0.10) | 0.13 (-0.75, 1.01) | -0.29 (-0.92, 0.34) | | -0.47 (-0.97, 0.03) | 1.13 (0.03, 2.24) | 0.01 |
| PM_10_ | -0.13 (-0.33, 0.06) | -0.01 (-0.45, 0.42) | -0.07 (-0.53, 0.40) | | -0.20 (-0.51, 0.11) | 0.29 (-0.67, 1.24) | 0.23 |
| ***Post-menopausal*** |  |  |  | |  |  |  |
| 1-year prior | n=1,634 | n=738 | n=360 | | n=274 | n=262 |  |
| PM_2.5_ | -0.18 (-0.54, 0.18) | -0.79 (-1.42, -0.16) | -0.14 (-1.24, 0.96) | | 0.01 (-0.60, 0.62) | 0.27 (-0.72, 1.26) | 0.48 |
| PM_2.5-10_ | -0.13 (-0.37, 0.11) | -0.69 (-1.42, 0.05) | 0.06 (-0.75, 0.87) | | 0.26 (-0.18, 0.71) | -0.86 (-1.83, 0.10) | 0.13 |
| PM_10_ | -0.12 (-0.30, 0.06) | -0.45 (-0.82, -0.08) | -0.01 (-0.55, 0.54) | | 0.11 (-0.18, 0.39) | -0.43 (-1.25, 0.39) | 0.16 |
| 3-year average | n=1,634 | n=745 | n=364 | | n=272 | n=253 |  |
| PM_2.5_ | -0.11 (-0.47, 0.25) | -0.76 (-1.41, -0.11) | -0.03 (-1.14, 1.08) | | 0.15 (-0.46, 0.76) | 0.19 (-0.81, 1.19) | 0.29 |
| PM_2.5-10_ | -0.09 (-0.33, 0.15) | -0.69 (-1.42, 0.05) | 0.15 (-0.65, 0.96) | | 0.30 (-0.13, 0.73) | -0.69 (-1.71, 0.33) | 0.15 |
| PM_10_ | -0.08 (-0.26, 0.10) | -0.43 (-0.81, -0.06) | 0.06 (-0.48, 0.61) | | 0.15 (-0.12, 0.43) | -0.35 (-1.20, 0.51) | 0.16 |

Abbreviations: U.S., United States; PM_2.5_, Particulate matter less than 2.5 microns in diameter; PM_2.5-10_, Particulate matter between 2.5 to 10 microns in diameter; PM_10_, Particulate matter less than 10 microns in diameter.

^a^Adjusted for cohort, age at mammogram, body mass index at mammogram, parity and age at 1^st^ birth categories, hormonal therapy use, history of biopsy-confirmed benign breast disease, and date of mammogram.

**Table S2.** Adjusted^a^ estimates of the difference in untransformed mammographic density measures for a 10 µg/m^3^ increase in PM using bootstrapped robust standard errors to estimate 95% CI

|  | Estimates of differences in Untransformed Mammographic Density Measures (95% CI) |
| --- | --- |
| ***Post-menopausal in the Northeast*** | n=745 |
| Percent Mammographic Density (Percentage points) |  |
| 3-year average PM_2.5_ | 3.4 (-0.5, 7.3) |
| Non-Dense Area (cm^2^) |  |
| 3-year average PM_2.5_ | -17.2 (-36.1, -0.5) |
| ***Post-menopausal in the West*** | n=272 |
| Percent Mammographic Density (Percentage points) |  |
| 3-year average PM_2.5-10_ | -2.0 (-4.7, 0.6) |

Abbreviations: PM_2.5_, Particulate matter less than 2.5 microns in diameter; PM_2.5-10_, Particulate matter between 2.5 to 10 microns in diameter

^a^Adjusted for cohort, age at mammogram, body mass index at mammogram, parity and age at 1^st^ birth categories, hormonal therapy use, history of biopsy-confirmed benign breast disease, and date of mammogram.
